# Giant pandas in captivity undergo short-term adaptation in nerve-related pathways

**DOI:** 10.1186/s40850-024-00195-y

**Published:** 2024-02-21

**Authors:** Yan Li, Wei Xu, Juan Wang, Hong Liu, Jiawen Liu, Liang Zhang, Rong Hou, Fujun Shen, Yuliang Liu, Kailai Cai

**Affiliations:** 1 Chengdu Research Base of Giant Panda Breeding, Panda Avenue, Northern Suburb, Chengdu, China; 2 Sichuan Key Laboratory of Conservation Biology On Endangered Wildlife, Panda Avenue, Northern Suburb, Chengdu, China

**Keywords:** Giant panda, Captivity, Adaptation, Chromatin structure

## Abstract

**Background:**

Behaviors in captive animals, including changes in appetite, activity level, and social interaction, are often seen as adaptive responses. However, these behaviors may become progressively maladaptive, leading to stress, anxiety, depression, and other negative reactions in animals.

**Results:**

In this study, we investigated the whole-genome sequencing data of 39 giant panda individuals, including 11 in captivity and 28 in the wild. To eliminate the mountain range effect and focus on the factor of captivity only, we first performed a principal component analysis. We then enumerated the 21,474,180 combinations of wild giant pandas (11 chosen from 28) and calculated their distances from the 11 captive individuals. The 11 wild individuals with the closest distances were used for the subsequent analysis. The linkage disequilibrium (LD) patterns demonstrated that the population was almost eliminated. We identified 505 robust selected genomic regions harboring at least one SNP, and the absolute frequency difference was greater than 0.6 between the two populations. GO analysis revealed that genes in these regions were mainly involved in nerve-related pathways. Furthermore, we identified 22 GO terms for which the selection strength significantly differed between the two populations, and there were 10 nerve-related pathways among them. Genes in the differentially abundant regions were involved in nerve-related pathways, indicating that giant pandas in captivity underwent minor genomic selection. Additionally, we investigated the relationship between genetic variation and chromatin conformation structures. We found that nucleotide diversity (θπ) in the captive population was correlated with chromatin conformation structures, which included A/B compartments, topologically associated domains (TADs) and TAD-cliques. For each GO term, we then compared the expression level of genes regulated by the above four factors (AB index, TAD intactness, TAD clique and PEI) with the corresponding genomic background. The retained 10 GO terms were all coordinately regulated by the four factors, and three of them were associated with nerve-related pathways.

**Conclusions:**

This study revealed that giant pandas in captivity undergo short-term adaptation in nerve-related pathways. Furthermore, it provides new insights into the molecular mechanism of gene expression regulation under short-term adaptation to environmental change.

**Supplementary Information:**

The online version contains supplementary material available at 10.1186/s40850-024-00195-y.

## Background

Over the past 15,000 years, the phenotype and genotype of multiple animal species, such as cattle, dogs, goats, horses, pigs and sheep, have been substantially altered during their adaptation to the human niche [1]. Long-term domestication and artificial selection have given rise to a substantial number of genomic structural variations, leading to a wide range of phenotypic diversity. Recent studies have identified numerous DNA variants, such as SNPs, InDels, and genomic structural variations, in the genome of ancestral species when compared to the corresponding chromosome-level reference assembly of domestic species. This indicates that the accumulation of several variations occurred during the long-term evolutionary history of adaptation [2–8]. These variations have the potential to impact gene expression by altering the sequence, epigenetic modification state, and chromatin spatial interaction of the target genes, ultimately leading to phenotypic diversity [9, 10]. This raises the question as to whether short-term adaptation could induce genomic variations, affecting gene expression by transcriptional control or other molecular mechanisms.

A recent study demonstrated that changes in the epigenetic state were accompanied by short-term adaptation to environmental stimulation [11]. The recent application of high-throughput chromatin conformation capture (Hi-C) technology revealed that the genomes of many species were organized into hierarchical chromatin structures that affect gene expression, including compartments [12], TADs [13], TAD-cliques [14] and promoter enhancer interactions (PEIs) [15]. Compartment A (accessible chromatin) enriched for both activating (H3K36 trimethylation) and repressing (H3K27 trimethylation) chromatin marks was open, accessible, actively transcribed chromatin [12, 16]. Disruption of TAD structures could lead to ectopic contacts and misexpression of genes, which could contribute to dramatic phenotypic changes [17–19]. Expansion of TAD-cliques was associated with transcriptional downregulation of genes and tended to occur in the nuclear periphery [14]. All of these chromatin structures contributed to the regulation of gene expression.

Giant pandas (*Ailuropoda melanoleuca*) have been kept in captivity since the late 1930s when the first successful capture of a live panda occurred. The captured cub, named Su Lin, was brought to the United States. Initially, captive pandas faced numerous challenges, and their survival rate was relatively low [20]. However, advancements in capturing techniques and an improved understanding of panda biology and care requirements have led to increased success in keeping them in captivity. Captive breeding programs aimed at increasing the panda population and its genetic diversity have gained prominence. These programs also sought to study the behavior, biology, and reproduction of giant pandas. Notable progress has been made in understanding their reproductive physiology, leading to successful breeding and the birth of panda cubs in captivity. The IUCN Red List has recently downgraded the panda from “endangered” to “vulnerable” in terms of extinction risk [21]. There are comparable numbers of captive and wild giant pandas [22], providing excellent resources for studying genome variations that might be induced by short-term adaptations related to habitat and diet. Evidence has already shown that the virome and gut microbiome are different between captive and wild giant pandas [23–25]. However, few studies have investigated genome variation due to short-term adaptation.

## Results

### Elimination of the population effect in the giant panda

To investigate the short-term adaptation of giant pandas in captivity, 535 Gb of high-quality data from 39 samples consisting of 11 captive and 28 wild giant pandas were downloaded from the National Center for Biotechnology Information (NCBI) database (Table S1). We then mapped the data to the genome (ASM200744v2); the detailed mapping information is provided in Table S2.

**Fig. 1:**
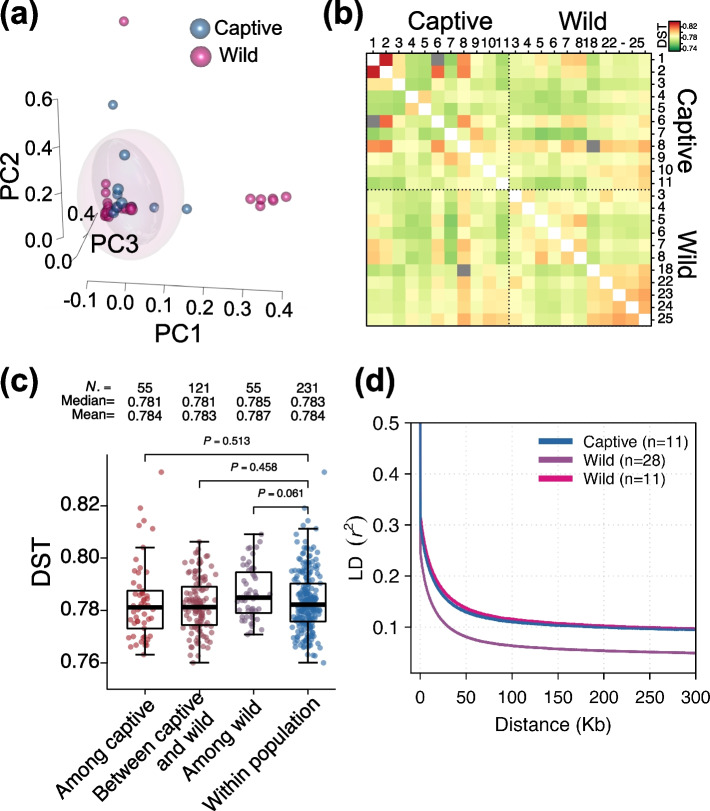
Workflow to eliminate population effect: **a** 3D scatter plot of the first three principle components of 39 individuals. Eclipse indicated the retained 11 giant pandas in captivity and wild; **b** Heatmap of DST distribution in the 22 individuals; **c** Comparison of relationship within or among the 4 groups; **d** LD patterns in the captive and wild population. The pink line indicated LD pattern of the selected 11 wild individuals for the subsequent analysis

Given that the giant panda was classified into five populations (Daxiangling, Liangshan, Minshan, Qionglai and Xiaoxiangling) and that the captive individuals were all hybrid individuals, we tried to choose enough wild individuals with the closest possible relationships to the 11 captive individuals. We first performed principal component analysis using PLINK and chose the top 3 components, which accounted for 27.78% of the total variation. We then enumerated all the possible combinations of wild individuals [11 chosen from 28] and calculated their distances from the 11 captive individuals. We finally confirmed 11 wild individuals for the subsequent analysis (Fig. 1a, Table S2). We used the DST (identical by state distance) to measure the relationship among populations and found no significant difference (*P* ≥ 0.05) when compared with the background (Fig. 1b and c). LD patterns further proved that there was almost no difference between captive and wild individuals (Fig. 1d), indicating that the population effect was eliminated as much as possible.

### Giant pandas in captivity underwent minor genomic selection on nerve-related pathways

**Fig. 2:**
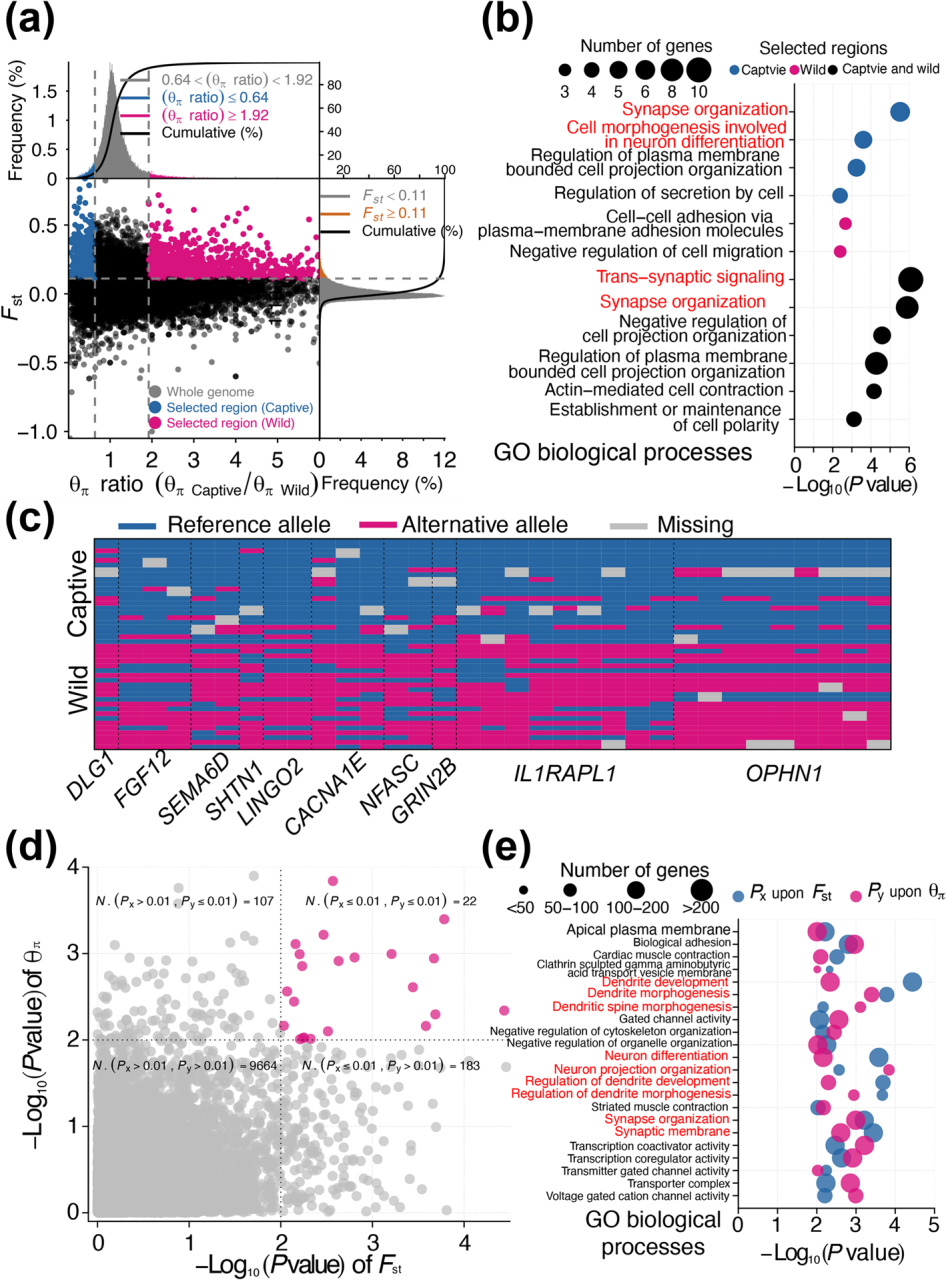
Genome selection analysis of giant panda in captivity: **a** Distribution of θ_π_ ratios (θ_π_, Captive/θ_π_, Wild) and F_ST_ values. The left and right vertical dashed lines corresponded to the 5% left and right tails of the empirical θ_π_ ratio distribution. The horizontal dashed line indicated the 5% right tail of the empirical F_ST_ distribution. The blue and red points were identified as selected regions for giant pandas in captivity and wild respectively; **b** GO enrichment analysis of the selected regions; **c** Distribution of the 32 SNPs allele frequency in the 10 nerve related pathways. The plot demonstrated the state of alleles in each locus among the 22 individuals; **d** Scatter plot of significance level, which calculated the F_ST_ or θ_π_ ratios of genes in each GO term between captive and wild population; **e** The retained 22 significant GO terms with *P* value ≤ 0.01 both upon F_ST_ and θ_π_

Based on the distribution of the nucleotide diversity (θ_π_) ratios (θ_π, Captive_/θ_π, Wild_) and F_ST_ values, 1,311 selected genomic regions were identified (Fig. 2a, Table S3). To exclude the effect that selected regions driven by genetic drift, we divided the 84 giant pandas in captivity into 5 generations (F1, F2, F3, F4 and F5) according to maternal inheritance and calculated θ_π_ in each generation. We found that there was no significant change among the genetic diversity of the five generations, indicating that the effect of the founder population was limited (Figure S1).

To reinforce the reliability of the selected regions, we retained 505 regions with at least one SNP showing an absolute frequency greater than 0.6 between the captive and wild populations (Figure S2).

Interestingly, GO analysis revealed that only the retained 34 selected genes from captive individuals belonged to these classes of genes that participated in synapse organization (GO:0050808, *P* = 2.97 × 10^–6^), cell morphogenesis involved in neuron differentiation (GO:0048667, *P* = 2.51 × 10^–4^) and other basic metabolic processes (Fig. 2b). The allele frequency of 32 SNPs in the 10 selected genes related to nerve pathways from captive individuals was significantly different between the captive and wild populations (Fig. 2c).

We then calculated the selection strength of genes associated with each GO term in relation to the θ_π_ and F_ST_ values. Out of the 22 significant GO terms, there were 10 nerve-related pathways, which firmly indicated that giant pandas in captivity underwent minor genomic selection (Fig. 2d and e). Additionally, the coverage of repeat elements (SINEs, LINEs and LTRs) in selected regions revealed significant differences from the whole genome (Figures S3 and S4). We then identified the regions with different sequencing depths between the captive and wild populations and found that the affected genes were also involved in nerve-related pathways (Figure S5). We also performed Genome-Wide Association Study (GWAS) by using the Plink software. The 11 giant pandas in captivity were defined as Case and the 28 giant pandas in the wild were defined as Control. Interestingly, we identified 648 SNPs under stringent cutoff (*P* ≤ 1 × 10^–7^) (Figure S6). GO analysis of the SNPs revealed that the most significant GO term was the cell morphogenesis involved in neuron differentiation, which further proved our finding (Figure S7).

### Nucleotide diversity (θ_π_) in the captive population was correlated with chromatin conformation structures

Chromatin conformation structures play a crucial role in the regulation of gene expression and the epigenetic control of cellular phenotype, leading us to investigate the relationship between genetic variation and these structures. We first investigated the relationship between chromatin accessibility and nucleotide diversity in captivity.

**Fig. 3:**
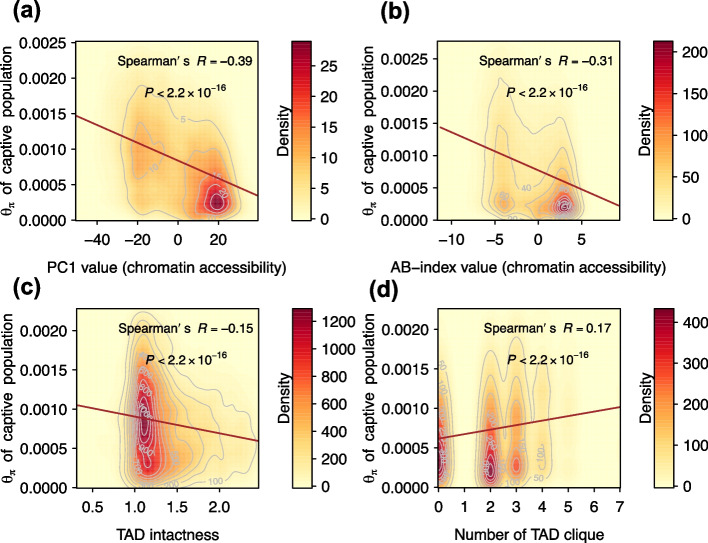
The 2D density heatmap between θ_π_ and hierarchical chromatin structures in captive population. The color scale represented the density of the data, with red indicating the highest density and yellow indicating the lowest density. The spearman correlation between the two variables was listed on the top of the plot: **a** PC1 value with 100 Kb resolution; **b** AB-index with 25 Kb resolution; **c** TAD intactness; **d** Number of TAD clique

Our research findings revealed that captive giant pandas have higher nucleotide diversity in closed chromatin regions. This was supported by a negative correlation between A/B compartments and θ_π_ (*R* = -0.39, *P* < 2.20 × 10^–16^), as well as a negative correlation between the AB-index value and θ_π_ (*R* = -0.31, *P* < 2.20 × 10^–16^) (Fig. 3a and b).

TAD intactness is a measure of the degree to which the 3D genomic structure of a cell's DNA remains undisturbed or preserved. We found that the intactness of TADs was negatively correlated with nucleotide diversity (*R* = -0.15, *P* < 2.20 × 10^–16^) (Fig. 3c), and the clique size of TADs was positively correlated with nucleotide diversity (*R* = 0.17, *P* < 2.20 × 10^–16^) (Fig. 3d). These results indicated a correlation between nucleotide diversity and chromatin conformation structures in captive giant pandas, suggesting that short-term captivity might have an impact on 3D chromatin conformation structures to some extent, leading to adaptation over time.

### Chromatin conformation structures coordinately regulate gene expression in nerve-related pathways

**Fig. 4:**
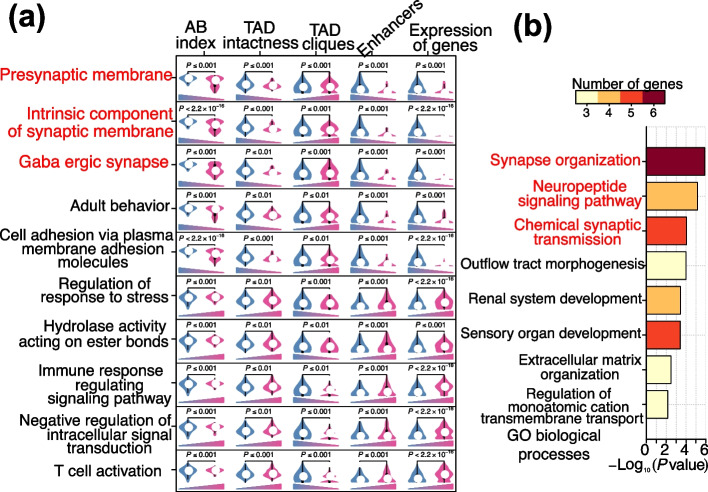
Identification of GO terms which were coordinately regulated by the chromatin structures in terms of AB-index, TAD, TAD-clique and PEI: **a** GO terms with 4 factors and expression of genes reaching the threshold of significance (*P* ≤ 0.01) when compared with genomic background. Left and right boxplot in each panel were genome background and genes in the GO term respectively. Triangle shape indicated the changing tendency; **b** GO analysis of the 34 functionally annotated genes filtered under the following criteria, top 5% of the TAD cliques and bottom 5% of the AB index, TAD intactness and enhancers

To determine the GO terms affected by chromatin conformation structures, we first constructed chromatin structures in terms of the AB-index, TAD, TAD-clique and PEI. We then compared the expression levels of genes regulated by the above four factors (AB index, TAD intactness, TAD clique and PEI) with the corresponding genome background for each GO term. We then retained the GO terms associated with all 4 factors and the genes whose expression reached the threshold of significance (*P* ≤ 0.01) (Fig. 4a). Genes related to the retained 10 GO terms were all coordinately regulated by the 4 factors (Fig. 4a).

Interestingly, we noted that 3 GO terms were related to nerve-related pathways (presynaptic membrane, intrinsic component of synaptic membrane and GABAergic synapse), and the genes associated with these GO terms were all downregulated (Fig. 4a). We further identified 34 functionally annotated genes under the following criteria: top 5% of the TAD cliques and bottom 5% of the AB index values, TAD intactness values and enhancers. These genes were mainly involved in nerve-related pathways, which included synapse organization, neuropeptide signaling pathway and chemical synaptic transmission (Fig. 4b). These results indicated that genes involved in nerve-related pathways might be coordinately regulated by chromatin conformation structures.

## Discussion

### Correlation between nucleotide diversity (θ_π_) and chromatin conformation structures

Nucleotide diversity may influence chromatin conformation through its effects on histone modifications, which play a critical role in regulating gene expression. For example, certain genetic variants have been shown to alter the positioning of nucleosomes, which can have a significant impact on gene expression [26]. Other studies have shown that nucleotide diversity can affect the binding of transcription factors to DNA, which can in turn impact chromatin conformation [27]. There is also evidence to suggest that chromatin conformation structures may influence nucleotide diversity. For example, recent studies have demonstrated that certain chromatin structures can create regions of reduced nucleotide diversity, possibly due to the effects of DNA methylation or other epigenetic modifications [28, 29]. Additionally, chromatin structure can impact the accessibility of different regions of DNA, potentially leading to differences in the frequency and distribution of genetic variants within a population [30].

Overall, the relationship between nucleotide diversity and chromatin conformation structures is complex and likely involves multiple mechanisms, including the impact of epigenetic modifications on chromatin structure and gene expression, as well as the effects of chromatin structure on nucleotide diversity. Understanding these dynamics has the potential to shed light on important questions related to gene regulation, evolution, and disease susceptibility.

### Effect of the 3D chromatin structure on gene expression

The enrichment of open chromatin was observed in A regions, while closed chromatin was highly associated with B regions, which corresponded to different levels of gene expression [31]. In the nuclear space, regions exhibiting similar transcriptional activity levels tended to colocalize, indicating a global tendency for euchromatin and heterochromatin to segregate [32]. The fundamental units of genome organization, known as topologically associating domains (TADs), consist of chromatin loops that facilitate promoter–enhancer interactions and regulate gene expression [33]. Interestingly, we discovered a significant correlation between the global interaction intensity of TADs and gene expression levels. Another level of genome organization, termed TAD cliques, involves long-range associations between TADs [14]. TAD cliques represent higher-order assemblies of repressed chromatin domains, which are predominantly enriched in B compartments as opposed to A compartments.

In our investigation, we made an interesting discovery of 2,885 TAD cliques, and their size exhibited an association with the downregulation of gene expression. Furthermore, we observed a negative correlation between the size of TAD cliques and the intactness of TADs. These findings suggest that gene expression is influenced by the hierarchical spatial organization of the genome, and we demonstrated that these units can collectively regulate gene expression. Indeed, by using a method that allows simultaneous profiling of chromatin architecture and gene expression, Liu et al. found that the establishment of specific chromatin interactions is tightly related to transcriptional control and cell functions during lineage specification [34]. This finding provides new insights into the relationship between 3D genome structures and the complex molecular processes underlying gene activation [34].

### Effect of short-term adaptation to captivity

Captive breeding for species of conservation concern involves bringing the endangered animals into captivity with the hope of rearing sustained captive populations for eventual reintroduction into the wild [35]. Captivity could lead to genetic change that might reduce a species’ ability to sustain itself once the population is reintroduced into the wild [36, 37]. In this study, we found that giant pandas in captivity experienced minor genomic selection, providing molecular evidence for genetic changes.

Furthermore, there were only 2,234 SNPs with an absolute frequency greater than 0.6 between the captive and wild populations, indicating that only a few of the genomic regions were under minor selection pressure in captivity. Although 861 (38.54%) SNPs were located in gene regions, only 2 SNPs were located in the coding sequence region, which included ARL14 (ADP ribosylation factor like GTPase 14) and SERPINB10 (serpin family B member 10). Interestingly, ARL14 was proven to control the movement of vesicles along the actin cytoskeleton in dendritic cells [38].

Almost all the SNPs were located in noncoding regions, suggesting that minor selection pressure (such as captivity of giant pandas) might affect gene expression by regulating neighboring noncoding regions. A GWAS identified a large number of genomic variants that were statistically associated with phenotypic variance [39–41]. For the noncoding region variants, the functional mechanism of causality was not clear. Recent studies have begun to focus on exploring the mechanism by which noncoding region variants control gene transcription by changing the spatial conformation of chromatin [42, 43]. Based on the evidence of giant pandas in captivity experiencing minor genomic selection in noncoding regions, it is necessary to compare the 3D chromatin organization between wild and captive giant pandas. This would provide an adequate explanation for the molecular basis of short-term adaptation to environmental change.

### Limited samples used for population genetics analysis

The population genetics analysis conducted in this study is subject to certain limitations due to the small sizes of both the wild and captive panda populations. These limitations could affect the interpretation and generalizability of the results.

First, small population sizes can lead to reduced genetic diversity [44]. In the case of wild pandas, the population has been severely fragmented and has experienced significant habitat loss. As a result, the genetic diversity within the wild population may be limited. This reduced genetic diversity can impact the accuracy of population genetics analyses, as there may be a limited number of genetic variations and alleles available for study. Consequently, the findings may not fully represent the broader genetic landscape of the species. Second, small population sizes can lead to increased genetic drift [45]. Genetic drift refers to the random fluctuations in allele frequencies that occur in small populations. In the case of pandas, both the wild and captive populations are relatively small, which increases the likelihood of genetic drift. Genetic drift can result in the loss of rare alleles and the fixation of certain alleles within the population. This can distort the genetic patterns observed and potentially affect the interpretation of results from population genetic analyses.

Moreover, the small sizes of the wild and captive populations may restrict the representativeness of the samples used for the analysis. It is important to ensure that the samples collected are representative of the overall population and include individuals from different geographic regions or subpopulations. However, for species with small populations, it may be challenging to obtain a diverse and representative sample, potentially introducing biases and limitations in the analysis.

## Conclusions

By comparing the WGS data of 11 captive and 28 wild giant pandas, 505 robust selected genomic regions were identified between the two populations. GO analysis revealed that genes in these regions were involved in nerve-related pathways. Additionally, nucleotide diversity in the captive population was correlated with chromatin conformation structures, which included A/B compartments, TADs and TAD-cliques. Chromatin conformation structures coordinately regulate gene expression in nerve-related pathways, further proving that giant pandas in captivity experience genomic selection on nerve-related pathways. In conclusion, we found that giant pandas in captivity underwent short-term adaptation in nerve-related pathways.

## Materials and methods

### Hi-C data processing pipeline

We used one-click pipeline for processing Hi-C datasets (Juicer). The pipeline first used BWA to map Hi-C reads to our giant panda genome. Duplicated and near-duplicate reads, reads that map to the same fragment, reads with low mapping quality (MAPQ < 30) were filtered. Contact matrices were then generated at various resolutions (10 Kb, 25 Kb, 100 Kb, 500 Kb and 1 Mb). The Normalized contact matrices were finally produced using KR algorithm.

### Resolution evaluation of Hi-C matrix

To evaluate the resolution of the Hi-C matrix in our study, we employed a systematic approach. Initially, we divided the genome into multiple window sizes, specifically 1 Kb, 2 Kb, 5 Kb, 10 Kb, 25 Kb, 40 Kb, 100 Kb, 500 Kb, and 1 Mb. Subsequently, we proceeded to count the number of Cis contacts within each window. Cis contacts were defined as any interaction where at least one read was mapped within the boundaries of the corresponding bin.

To determine the optimal resolution for our Hi-C matrix, we sought a window size that captured a significant proportion of interactions. Specifically, we calculated the percentages of bins with contacts greater than 1000 under multiple window sizes. The minimum window size with the percentage greater than 80 was defined as the Hi-C matrix resolution.

### Identification of compartment A/B at resolution of 100 Kb and 25 Kb

The compartment A/B analysis in our study was conducted using two different resolutions: 100 Kb and 25 Kb. At the 100 Kb resolution, we followed a previously described approach. Initially, a Pearson correlation matrix was generated using the 'cor' function in R. Then, we obtained the first three principal components using the 'prcomp' function on the correlation matrix. For compartment A/B classification at the 100 Kb resolution, we focused on the Spearman's correlation between PC1 values and gene density. Bins at the 100 Kb resolution showing a positive correlation were categorized as compartment A, while those without a positive correlation were categorized as compartment B.

To further explore the genomic organization at a higher resolution, we identified AB-index value at 25 Kb resolution. This analysis involved utilizing the A-B index value, which represented the comparative likelihood of a sequence interacting with compartment A or B at the 100 Kb resolution. At the 25 Kb resolution, bins with positive AB-index values, indicating a stronger association with compartment A at the 100 Kb resolution, were classified as A regions (25 Kb). Conversely, bins with negative AB-index values, indicating a stronger association with compartment B at the 100 Kb resolution, were classified as B regions (25 Kb).

### TAD identification

To identify topologically associated domains (TADs) in our study, we utilized the normalized contact matrix at a resolution of 25 Kb. TAD identification was performed using the directionality index (DI) score and a Hidden Markov Model (HMM), following the previously described method. We employed TADtools software with default parameters for this analysis. After the TADs were identified, we assessed their intactness as the previously established method. The calculation of TAD intactness was carried out using custom scripts specifically.

### TAD clique identification

To identify TAD cliques in our analysis, we followed a previously established methodology. Initially, we calculated the probability of observed and expected Hi-C contacts for each pair of TADs. This step allowed us to quantify the interactions between TADs. To select significant TAD-TAD interactions, we applied the Benjamini–Hochberg method with a false discovery rate threshold of < 0.01%. This stringent criterion ensured that only highly significant interactions were considered for further analysis. To determine the maximal TAD clique sizes for each TAD, we employed the 'find_cliques' method, which utilized the Bron-Kerbosch algorithm. This algorithm efficiently identified the largest cliques within the TAD network, providing valuable insights into the organization of TAD interactions.

### PEI identification

Based on the normalized contact matrix at a resolution of 10 Kb, we employed the PSYCHIC software to generate raw pairwise enhancer interactions (PEIs) [15]. Subsequently, we applied a filtering step to exclude low-confidence PEIs with an interaction distance lower than 60 Kb. By employing PSYCHIC and applying the distance-based filtering criterion, we ensured that only high-confidence PEIs were considered for further analysis. This approach allowed us to focus on robust enhancer interactions and explore their functional implications in the regulatory landscape of our study.

### Gene expression quantification

To estimate gene-level expression levels, we employed Kallisto, a high-speed transcript quantification tool. The expression levels were quantified in transcripts per million (TPM), which provides a normalized measure of gene expression accounting for both the gene length and sequencing depth.

### Functional enrichment analysis

To gain functional insights of our data, we conducted a comprehensive functional enrichment analysis of Gene Ontology (GO) terms and pathways. This analysis was performed using the A Gene Annotation & Analysis Resource, also known as Metascape (https://www.metascape.org/). During the analysis, we focused on Gene Ontology Biological Process (GO-BP), Gene Ontology Molecular Function (GO-MF), and KEGG pathway enrichment. To determine significant enrichment, we considered statistical significance terms and pathways with the *P*-value less than 0.05.

### SNP calling

The high-quality paired-end reads of 39 giant panda individuals were mapped to the reference genome (ASM200744v2) with average coverage depth 5 × for each individual using BWA software. SNPs were then called independently by GATK. SNPs with genotype quality < 30 or read depth < 50 were then filtered.

### Sample selection for the wild individuals

To minimize effect of population factor and focus on the survival environment (Captive and Wild), we first performed PCA analysis on all the 39 giant panda individuals (28 wild and 11 captive) using PLINK. We then calculated the euclidean distance of the first three principle components between any two pairs. Out of the 28 wild individuals, 11 with the closest distance to the 11 captive individuals were selected for the subsequent analysis.

### LD analysis

To evaluate LD decay, the squared correlation (*r*^*2*^) between any two loci was calculated using PopLDdecay with default parameter.

### Calculation of θ_π_ and FST

A sliding-window approach (40-kb windows sliding in 20-kb steps) was applied to quantify polymorphism levels (θ_π_, pairwise nucleotide variation as a measure of variability) and genetic differentiation (F_ST_) between captive and wild giant pandas using VCFtools.

### Identification of selected regions

To detect regions with significant signatures of selective sweep, we considered the distribution of the θ_π_ ratios (θ_π_, Captive/θ_π_, Wild) and F_ST_ values as described previously. We then calculate all the SNP frequency in captive and wild individuals respectively and kept SNPs with absolute difference ≥ 0.6. Finally, genes both in genome selected regions and harboring at least 1 SNP were retained for functional enrichment analysis.

### Supplementary Information

**Additional file 1: Table S1.** Summary of downloaded sample information used in this study.

**Additional file 2: Table S2.** Summary of the mapping information.

**Additional file 3: Table S3.** Summary of genomic selected regions in 40 Kb bins with 20 Kb step between Captive and Wild born giant pandas.

**Additional file 4:** **Figure S1. **θ_π_distribution of the five generations. **Figure S2. **Distribution of the 2,234 SNPs with absolute SNP frequency greater than 0.6 between captive and wild population. **Figure S3. **Comparison of GC content between genome selected region and whole genome. **Figure S4. **Comparison of different type of repeat elements coverage between genome selected region and whole genome. **Figure S5. **GO enrichment analysis of genes located in the differentially depth regions between captive and wild population. **Figure S6. **Manhattan plot of the SNPs across the whole genome. **Figure S7. **GO enrichment analysis of the 648 SNPs which distributed in 64 genes. 

## Data Availability

The following supporting information can be downloaded at: https://github.com/melady12/panda-genomics/tree/master/Supplementary%20Materials, Figure S1: θπ distribution of the five generations; Figure S2: Distribution of the 2,234 SNPs with absolute SNP frequency greater than 0.6 between captive and wild population. Plot in the top indicated number of SNPs across each chromosome. The middle plot demonstrated the state of alleles in each locus among the 22 individuals. The bottom plot was the distribution of frequency of alternative allele in captive and wild population respectively; Figure S3: Comparison of GC content between genome selected region and whole genome; Figure S4: Comparison of different type of repeat elements coverage between genome selected region and whole genome; Figure S5: GO enrichment analysis of genes located in the differentially depth regions between captive and wild population; Figure S6: Manhattan plot of the SNPs across the whole genome. The x-axis represented the length of chromosome and y-axis represented the log transformed *P* value; Figure S7: GO enrichment analysis of the 648 SNPs which distributed in 64 genes; Table S1: Summary of downloaded sample information used in this study; Table S2: Summary of the mapping information; Table S3: Summary of genomic selected regions in 40 Kb bins with 20 Kb step between captive and wild giant pandas. The 39 whole genome sequencing data were downloaded in SRA database (SRR504857, SRR504859, SRR504860, SRR504862, SRR504863, SRR504864, SRR504865, SRR504866, SRR504867, SRR504869, SRR504870, SRR504871, SRR504872, SRR504873, SRR504874, SRR504876, SRR504877, SRR504878, SRR504880, SRR504881, SRR504882, SRR504883, SRR504884, SRR504885, SRR504886, SRR504887, SRR504888, SRR504889, SRR504892, SRR504893, SRR504894, SRR504896, SRR504897, SRR504899, SRR504900, SRR504901, SRR504902, SRR504903, SRR504904) from NCBI (https://www.ncbi.nlm.nih.gov/). The detailed information was listed in Table S1.

## Abbreviations

GWAS: Genome-wide association study
GO: Gene ontology
Hi-C: High-throughput chromatin conformation capture
IUCN: International union for conservation of nature
LD: Linkage disequilibrium
LINE: Long interspersed nuclear element
LTR: Long terminal repeat
NCBI: National center for biotechnology information
PCA: Principal components analysis
PEI: Promotor enhancer interaction
SINE: Short interspersed nuclear element
SNP: Single nucleotide polymorphism
TAD: Topologically associated domain
WGS: Whole genome sequencing
