# Supplementary material for: Giant pandas in captivity undergo short-term adaptation in nerve-related pathways
Source: BMC Zool. 2024 Feb 21;9:4. doi: 10.1186/s40850-024-00195-y (PMC10880213; doi:10.1186/s40850-024-00195-y)
Supplement: Supplementary file 4 — Additional file 4: Figure S1. θπdistribution of the five generations. Figure S2. Distribution of the 2,234 SNPs with absolute SNP frequency greater than 0.6 between captive and wild population. Figure S3. Comparison of GC content between genome selected region and whole genome. Figure S4. Comparison of different type of repeat elements coverage between genome selected region and whole genome. Figure S5. GO enrichment analysis of genes located in the differentially depth regions between captive and wild population. Figure S6. Manhattan plot of the SNPs across the whole genome. Figure S7. GO enrichment analysis of the 648 SNPs which distributed in 64 genes. [file 40850_2024_195_MOESM4_ESM.docx]

**Supplementary Figures**


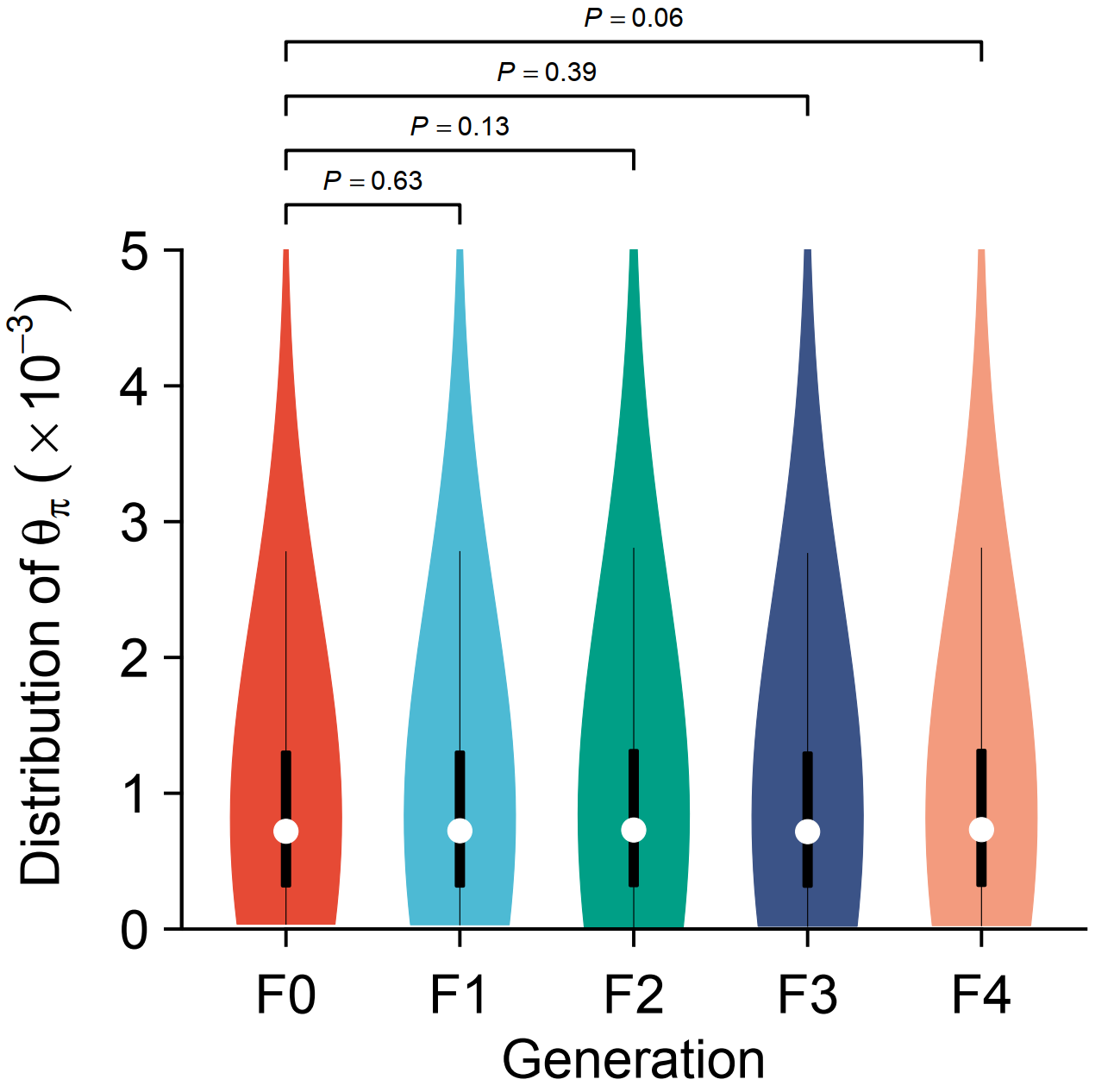


**Figure S1: θ_π_ distribution of the five generations.**


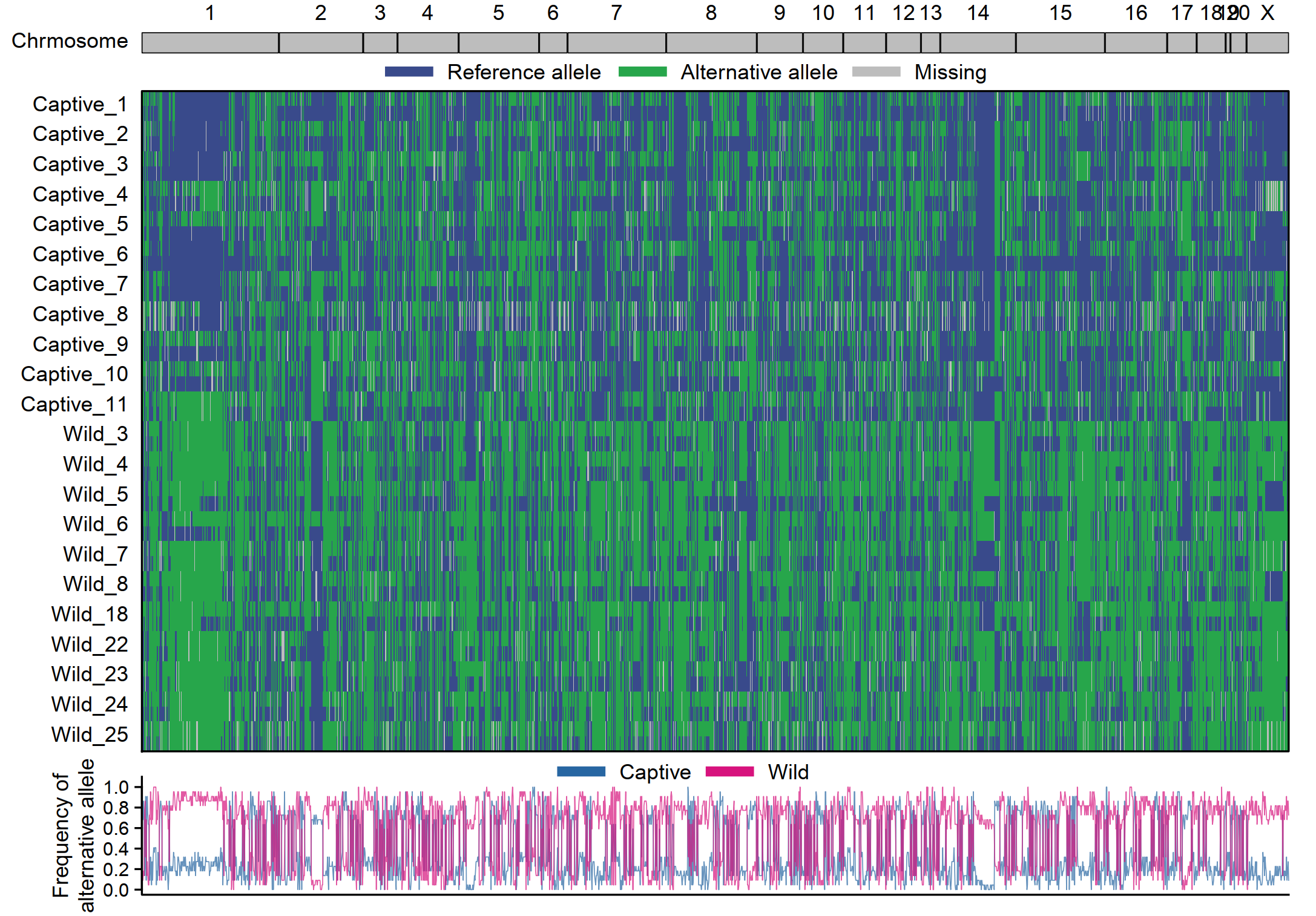


**Figure S2: Distribution of the 2,234 SNPs with absolute SNP frequency greater than 0.6 between captive and wild population.** Plot in the top indicated number of SNPs across each chromosome. The middle plot demonstrated the state of alleles in each locus among the 22 individuals. The bottom plot was the distribution of frequency of alternative allele in captive and wild population respectively.


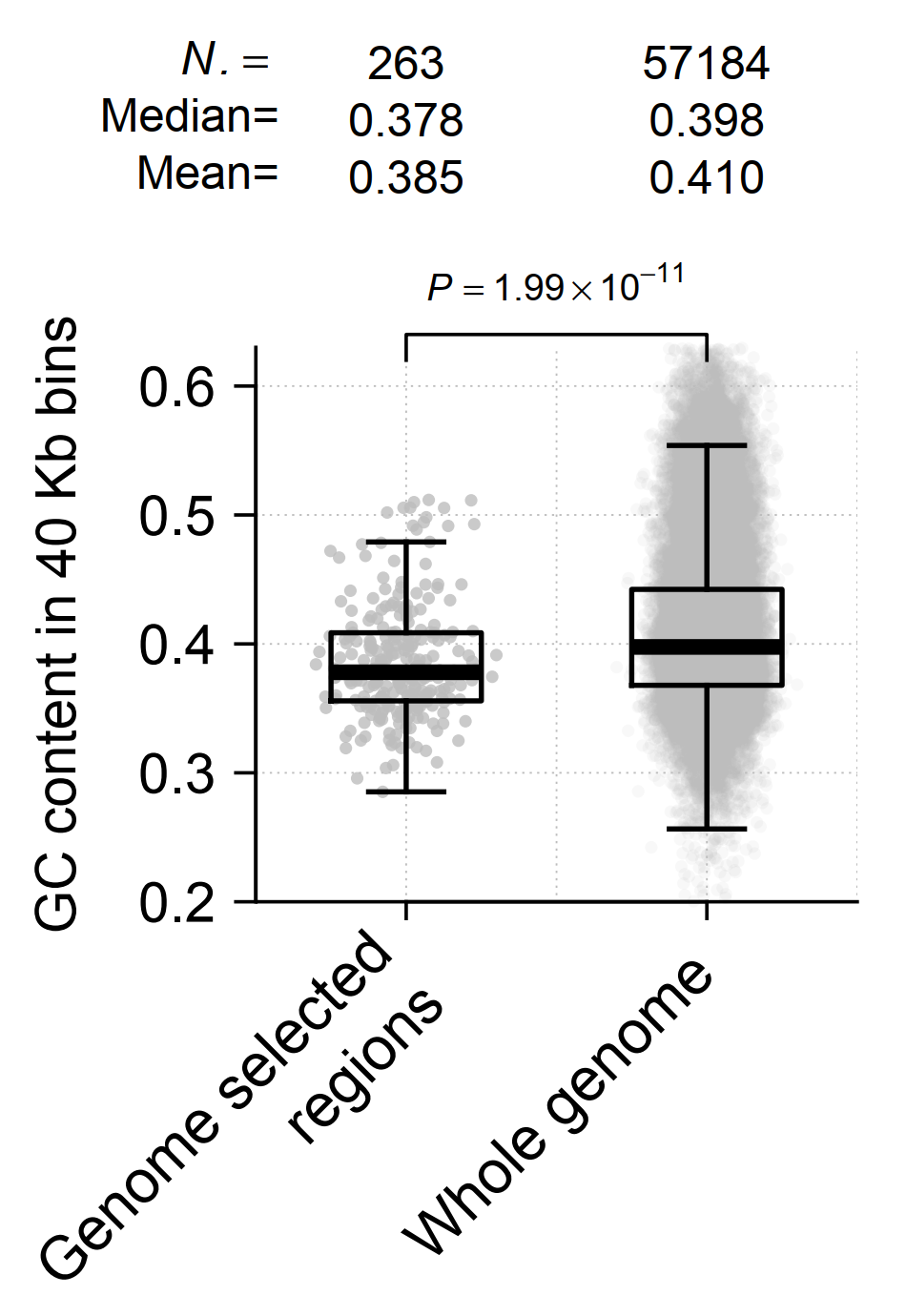


**Figure S3: Comparison of GC content between genome selected region and whole genome.**

**
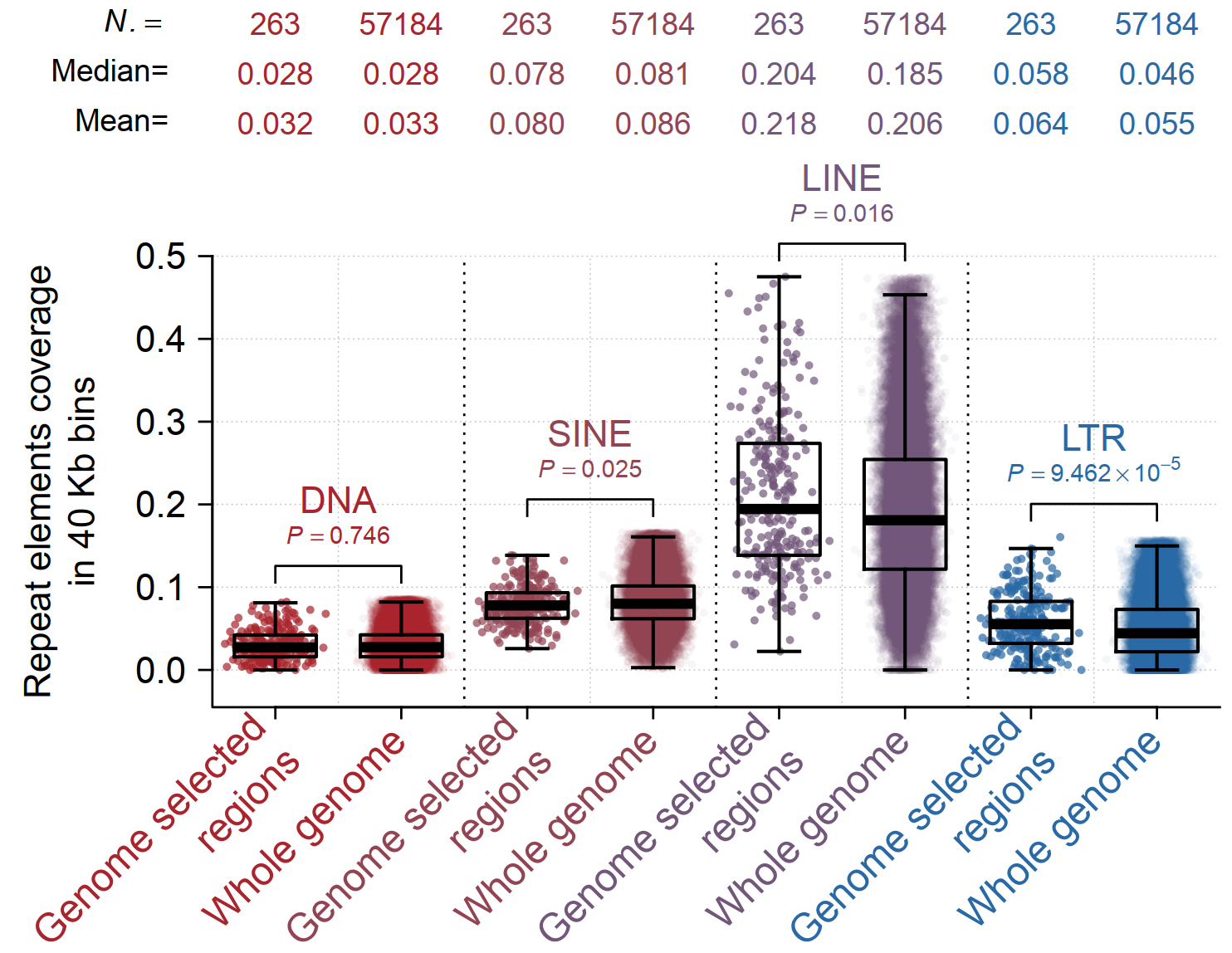
**

**Figure S4: Comparison of different type of repeat elements coverage between genome selected region and whole genome.**

**
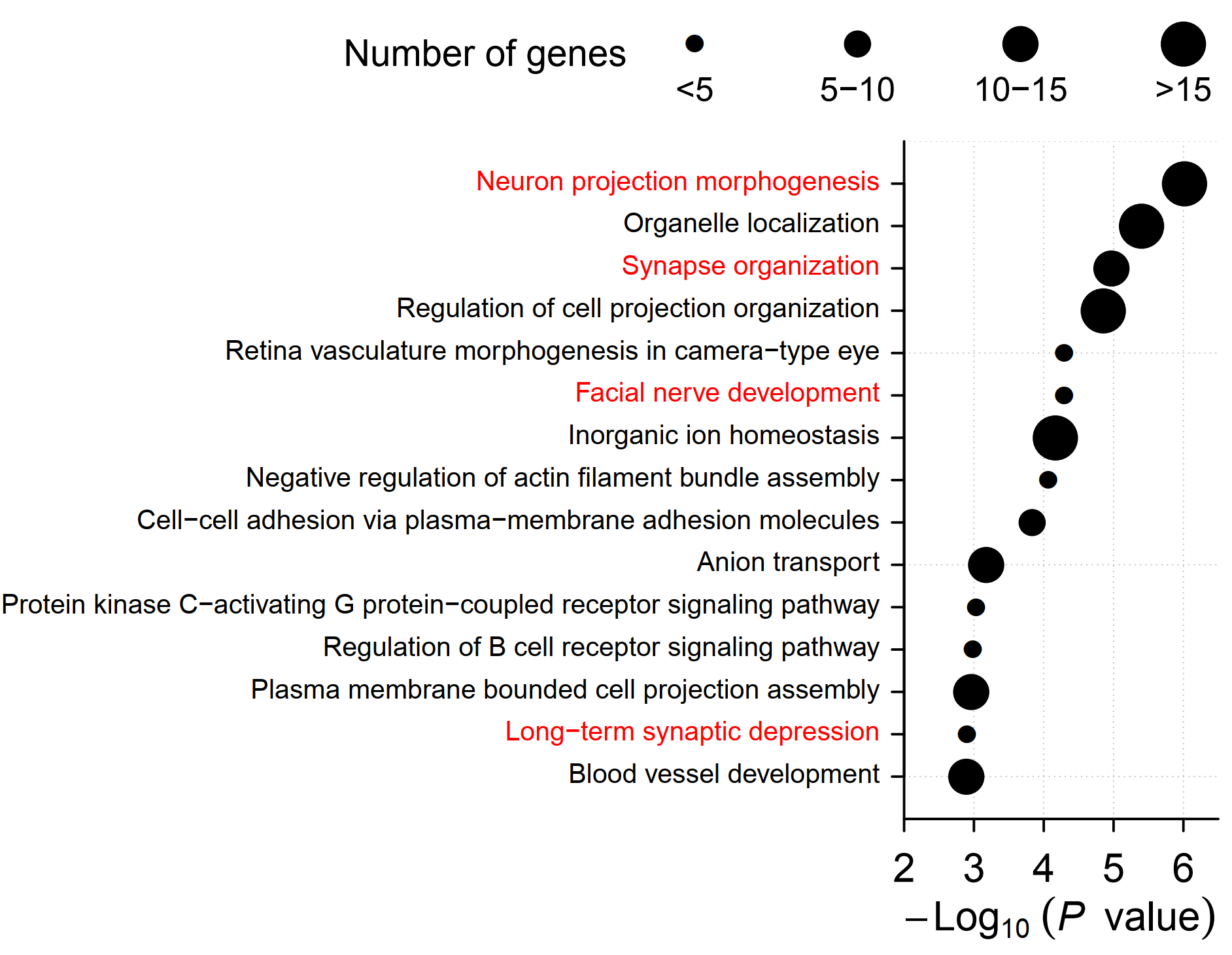
**

**Figure S5: GO enrichment analysis of genes located in the differentially depth regions between captive and wild population.**


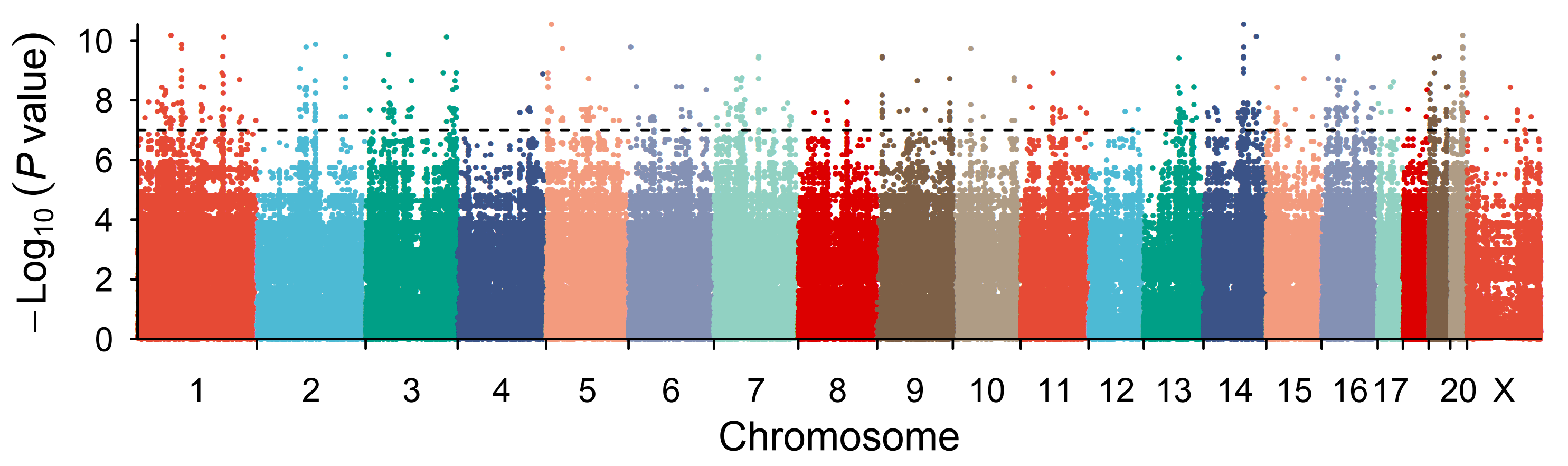


**Figure S6: Manhattan plot of the SNPs across the whole genome.** The x-axis represented the length of chromosome and y-axis represented the log transformed P value.


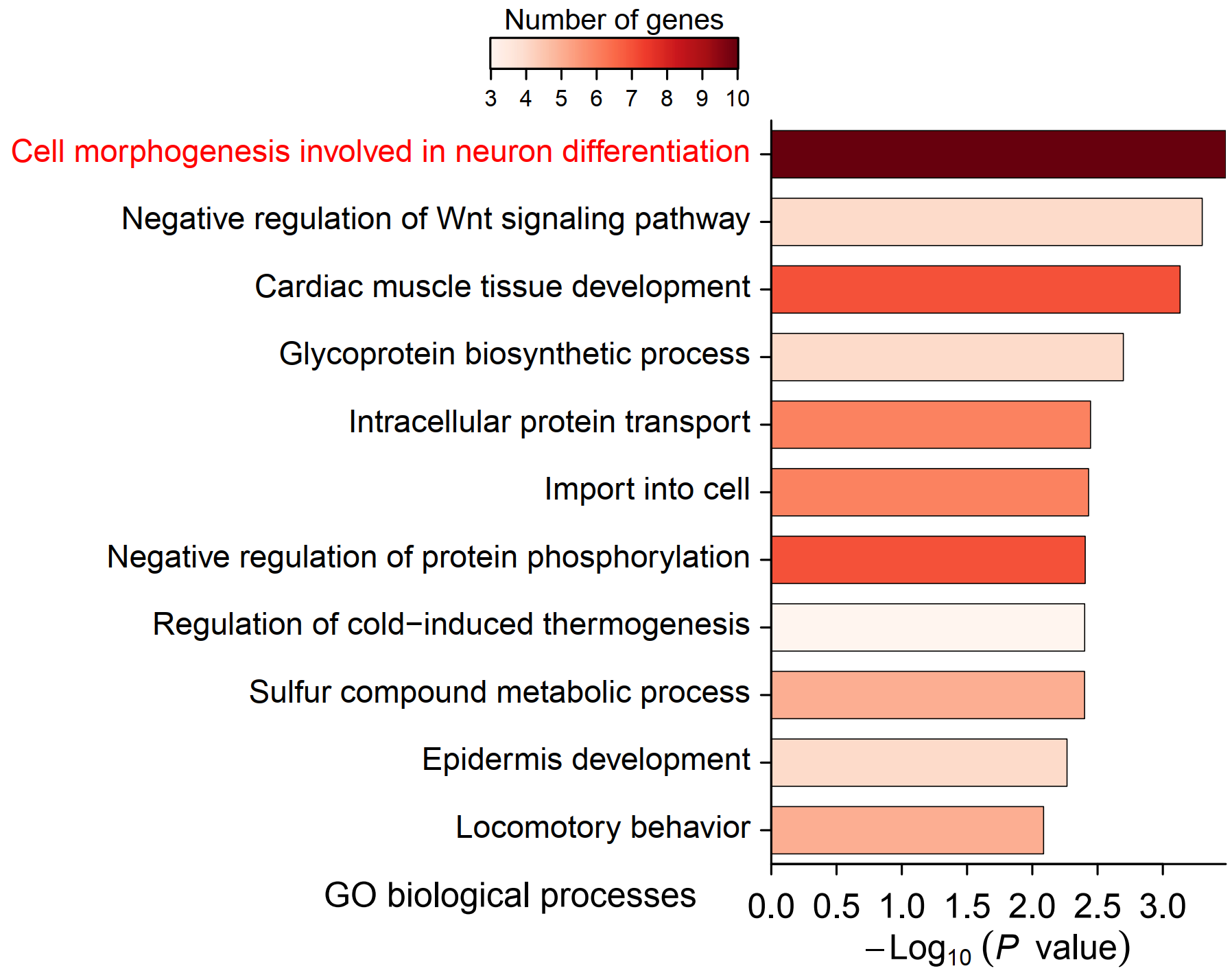


**Figure S7: GO enrichment analysis of the 648 SNPs which distributed in 64 genes.**
